# Ginsenoside Rb1 Attenuates Triptolide-Induced Cytotoxicity in HL-7702 Cells *via* the Activation of Keap1/Nrf2/ARE Pathway

**DOI:** 10.3389/fphar.2021.723784

**Published:** 2022-01-03

**Authors:** Hulinyue Peng, Longtai You, Chunjing Yang, Kaixin Wang, Manting Liu, Dongge Yin, Yuchen Xu, Xiaoxv Dong, Xingbin Yin, Jian Ni

**Affiliations:** ^1^ School of Chinese Materia Medica, Beijing University of Chinese Medicine, Beijing, China; ^2^ Department of Pharmacy, Beijing Shijitan Hospital Affiliated to Capital University of Medical Sciences, Beijing, China

**Keywords:** ginsenoside Rb1, triptolide, HL-7702 cells, Keap1/Nrf2/ARE pathway, apoptosis, cytotoxicity

## Abstract

Triptolide (TP) is the major bioactive compound extracted from *Tripterygium wilfordii* Hook F. It exerts anti-inflammatory, antirheumatic, antineoplastic, and neuroprotective effects. However, the severe hepatotoxicity induced by TP limits its clinical application. Ginsenoside Rb1 has been reported to possess potential hepatoprotective effects, but its mechanism has not been fully investigated. This study was aimed at investigating the effect of ginsenoside Rb1 against TP-induced cytotoxicity in HL-7702 cells, as well as the underlying mechanism. The results revealed that ginsenoside Rb1 effectively reversed TP-induced cytotoxicity in HL-7702 cells. Apoptosis induced by TP was suppressed by ginsenoside Rb1 *via* inhibition of death receptor-mediated apoptotic pathway and mitochondrial-dependent apoptotic pathway. Pretreatment with ginsenoside Rb1 significantly reduced Bax/Bcl-2 ratio and down-regulated the expression of Fas, cleaved poly ADP-ribose polymerase (PARP), cleaved caspase-3, and -9. Furthermore, ginsenoside Rb1 reversed TP-induced cell cycle arrest in HL-7702 cells at S and G2/M phase, *via* upregulation of the expressions of cyclin-dependent kinase 2 (CDK2), cyclin E, cyclin A, and downregulation of the expressions of p53, p21, and p-p53. Ginsenoside Rb1 increased glutathione (GSH) and superoxide dismutase (SOD) levels, but decreased the reactive oxygen species (ROS) and malondialdehyde (MDA) levels. Pretreatment with ginsenoside Rb1 enhanced the expression levels of nuclear factor-erythroid 2-related factor 2 (Nrf2), total Nrf2, NAD(P)H: quinone oxidoreductases-1 (NQO-1), heme oxygenase-1 (HO-1), and Kelch-like ECH-associated protein 1 (Keap1)/Nrf2 complex. Therefore, ginsenoside Rb1 effectively alleviates TP-induced cytotoxicity in HL-7702 cells through activation of the Keap1/Nrf2/ARE antioxidant pathway.

## 1 Introduction

**FIGURE 1:**
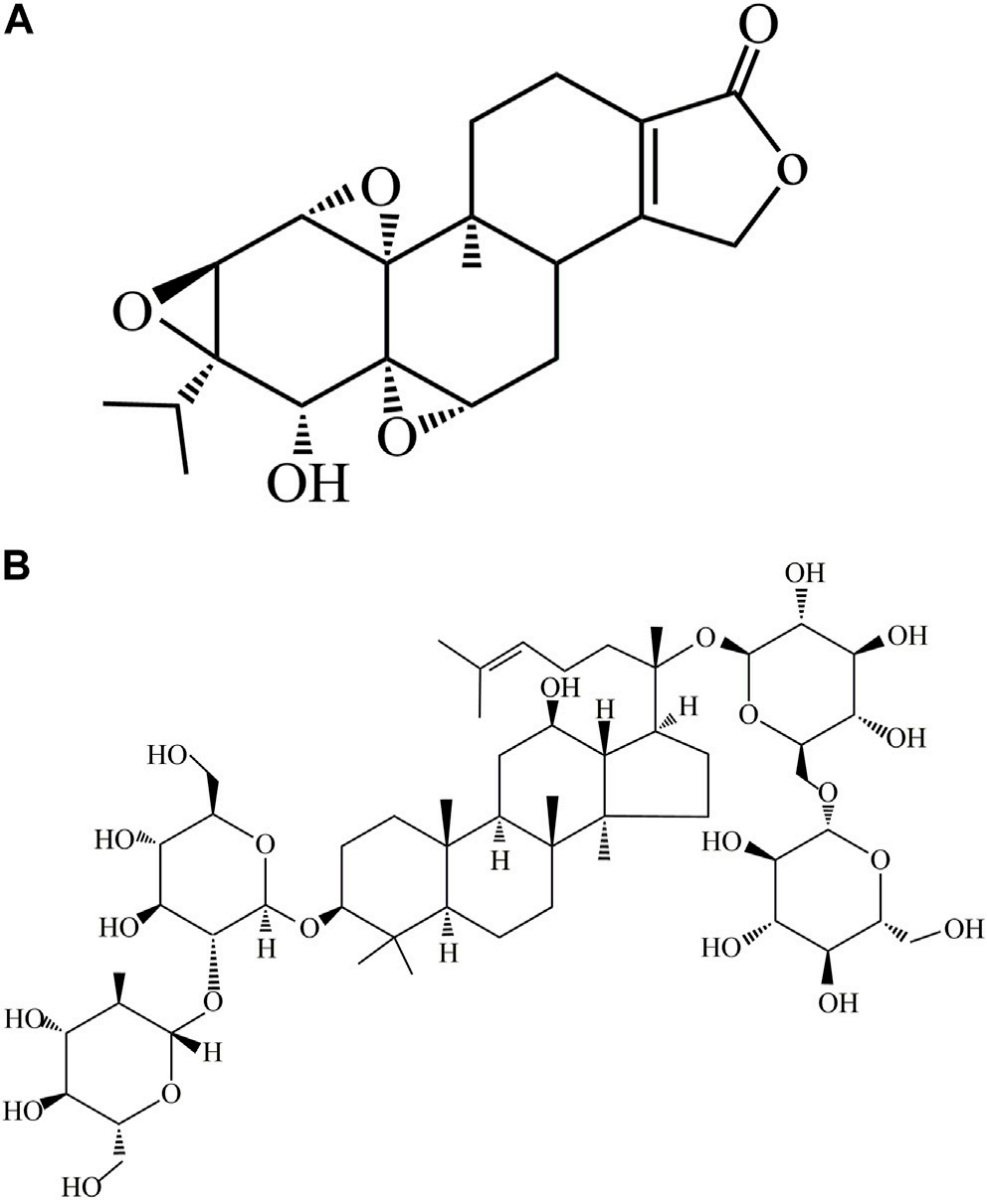
Chemical structures of ginsenoside Rb1 and TP. **(A)** Chemical structure of TP. **(B)** Chemical structure of ginsenoside Rb1.

Triptolide (TP) (Figure 1A) is a major diterpenoid extracted from *Tripterygium wilfordii* Hook F (TWHF), a rhizome used as a traditional Chinese medicinal herb (Tan et al., 2018). Studies have revealed that TP exerts significant pharmacological effects such as antirheumatic, anti-inflammatory, antineoplastic, and neuroprotective properties. These properties account for its wide application in the treatment of systemic lupus erythematosus, rheumatoid arthritis, central nervous system diseases, and nephrotic syndrome (Li et al., 2014). However, the clinical application of TP is hampered by its toxic effects on multiple cells and organs: it has been associated with nephrotoxicity, hepatotoxicity, cardiotoxicity, and reproductive toxicity (Ruan et al., 2017; You et al., 2018; Wang et al., 2019a; Xu et al., 2019b). The hepatotoxicity of TP is of widely concern since it may cause liver damage in animals and humans (Zhou et al., 2017a).

Oxidative stress-induced apoptosis and hepatocyte dysfunction are thought to be involved in the potential mechanisms underlying TP-induced hepatotoxicity (You et al., 2018). It has been reported that TP stimulated increases in levels of intracellular reactive oxygen species (ROS) and oxidative stress (Lee et al., 2015). Overproduction of intracellular ROS impairs the structure of Kelch-like ECH-associated protein 1 (Keap1)/nuclear factor-erythroid 2-related factor 2 (Nrf2) complex, resulting in activation Nrf2 which then binds to antioxidant response elements (ARE) in the nucleus. The Nrf2/ARE complex stimulates the transcription and translation of oxidoreductases such as heme oxygenase-1 (HO-1), glutamate cysteine ligase catalytic (GCLC), and NAD(P)H: quinone oxidoreductases-1 (NQO-1), which play important roles in antioxidant defense (Ci et al., 2017; Tan et al., 2018; Cinegaglia et al., 2020). As a result, the activation of Nrf2 pathway effectively regulates the levels of ROS and attenuates cell damage caused by oxidative stress.

Apoptosis is a physiological phenomenon which is initiated under the regulation of a series of genes when cells are exposed to drug or external stimuli (Garcia-Caballero et al., 2017; Nori-Garavand et al., 2020). One of the main regulatory pathways of apoptosis is death receptor-mediated pathway, also known as extrinsic pathway. Fas is an important death receptor that mediates apoptosis. The interaction between Fas and its ligand FasL activates caspase-8, thereby triggering activation of downstream effector caspase-3, and ultimately initiating apoptosis (Zhou et al., 2017b; Xu et al., 2019b; You et al., 2019). Another major pathway for regulation of apoptosis is the mitochondrial-dependent intrinsic apoptosis pathway. A key step in the intrinsic apoptosis pathway involves increased mitochondrial membrane permeability which leads to increased release of cytochrome *c* into the cytoplasm. Under oxidative stress, the excessive ROS interact with Bax, thereby enhancing the release of cytochrome *c* from the mitochondria. In the cytoplasm, cytochrome *c* forms an apoptotic body through interaction with caspase-9, which further stimulates the expression of downstream effector caspase-3, and ultimately initiates apoptosis (Li et al., 2016; Pan et al., 2018; Cao et al., 2019; Li et al., 2019). Studies *in vivo* and *in vitro* have shown that TP induces apoptosis through these two pathways (You et al., 2018; Huo et al., 2019; Hasnat et al., 2020).

Ginsenoside Rb1 (Figure 1B) is a dammarane-type triterpene saponin compound isolated from the widely used Chinese herb *Panax notoginseng* (Burk.) F. H. Chen (*P. notoginseng*) (Yu et al., 2019). It exerts significant pharmacological effects such as antioxidant, anti-inflammatory, cardioprotective, and neuroprotective properties (Zhu et al., 2012; Zhang et al., 2018; Jiang et al., 2021; Wang et al., 2021; Wei et al., 2021). Although the mechanism involved in the use of ginsenoside Rb1 for the treatment of certain diseases has been gradually understood over the past few years, its hepatoprotective potential has not been elucidated (Na et al., 2012; Lai et al., 2021). Therefore, this study was aimed at investigating the protective effect of ginsenoside Rb1 against TP-induced cytotoxicity, and its underlying mechanism, so as to provide new strategies for the intervention and treatment of hepatotoxicity caused by TP.

## 2 Materials and Methods

### 2.1 Reagents and Chemicals

The HL-7702 cells were purchased from China Infrastructure of Cell Line Resources. TP (batch no. 7675; purity: 99.7%), and ginsenoside Rb1 (batch no. 8786; purity: 100.0%) were purchased from Standard Technology Co., Ltd. (Shanghai, China). Fetal bovine serum (FBS), penicillin and streptomycin solution, and Dulbecco’s Modified Eagle’s Medium (DMEM) were purchasedfrom Corning (NY, United States). Phosphate buffered saline (PBS) and 0.05% trypsin were purchased from Gibco, Invitrogen (Carlsbad, CA, United States). 3-(4,5-Dimethylthiazol-2-yl-)-2,5-diphenyltetrazolium bromide (MTT) (ST316), N-acetylcysteine (NAC) (ST1546), LDH assay kits (C0017), malondialdehyde (MDA) kits (S0131S), GSH kits (S0053), TdT-mediated dUTP Nick-End Labeling (TUNEL) analysis kits (S1098), ROS detection kits (S0033S), SOD kits (S0087), bicinchoninic acid (BCA) protein quantification kits (P0012), dimethyl sulfoxide (DMSO), and JC-1 kits (C2003S) were products of Beyotime Biotechnology (Shanghai, China). Antibodies against Nrf2 (ab62352), Bax (ab53154), COX IV (ab16056), p53 (ab241556), Keap1 (ab118285), CDK2 (ab32147), cyclin A (ab181591), Bcl-2 (ab185002), Fas (ab82419), cyclin E (ab33911), cleaved caspase-3 (ab2302), cytochrome *c* (ab90529), p21 (188224), NQO1 (ab80588), β-actin (ab8226), cleaved caspase-9 (ab2324), and Histone H3 (ab1791) were purchased from Abcam (Cambridge, United Kingdom). Antibodies against cleaved PARP (5625S), HO-1 (86806S), Keap1 (4678S), and p-p53 (9286S) were purchased from Cell Signaling Technology, Beverly (MA, United States). ML385 (SML 1833) was purchased from Sigma-Aldrich (St. Louis, MO, United States).

### 2.2 Cell Cultures and Treatment

The HL-7702 cells were cultured in DMEM containing 1% penicillin/streptomycin and 10% FBS in a humidified 5%-CO_2_ incubator with at 37°C. Ginsenoside Rb1 and TP were dissolved in DMSO and preserved at 4°C. In all experiments, DMSO concentration was always kept below 0.1%. The HL-7702 cells were incubated, with or without pretreatment with ginsenoside Rb1 for 6 h, prior to treatment with 400 nM TP for 48 h. In this study, cells in logarithmic growth phase were used in all experiments.

### 2.3 Cell Viability Assay

The cells were seeded overnight in 96-well plates at a density of 5.0 × 10^3^ cells/well. Then, cells in pretreatment groups were exposed to 5–20 μM ginsenoside Rb1 for 6 h. Subsequently, the cells were exposed to TP (400 nM) for 48 h. Thereafter, 100 μL of MTT (0.5 mg/ml) working solution was added to the cells, followed by incubation for 4 h. Thereafter, the MTT medium was removed, and 150 μL of DMSO was added to the wells in order to dissolve the resultant formazan crystals. Then, the absorbance of the formazan solution was read at 490 nm in a microplate reader (Thermo Fisher Scientific, NYC, United States).

### 2.4 LDH Cytotoxicity Assay

The cells were seeded in 96-well plates at a density of 1.0 × 10^4^ cells/well, and they were cultured in DMEM overnight. Subsequent incubation was carried out as described in Section 2.2. The activity of LDH in the supernatant was assayed using LDH assay kit.

### 2.5 TUNEL Staining

The cells were seeded in 6-well plates at a density of 4.0 × 10^5^ cells/well. After exposure to ginsenoside Rb1 and TP or TP alone, the cells in the different groups were treated with 4% paraformaldehyde solution. Then, TUNEL analysis kit was used to determine apoptosis according to the instructions in the kit. Apoptotic cells were identified and photographed under a florescence microscope (BX51-DSU; Olympus, Tokyo, Japan) at ×200 magnification.

### 2.6 Apoptosis Assay

The cell culture procedure used was as described in Section 2.2. After washing with PBS, the cells were re-suspended in 295 μL binding buffer, followed by incubation with. Annexin V-FITC and propidium iodide for 20 min at room temperature, in the dark. Thereafter, the cells were injected into a flow cytometer (BD Biosciences, New Jersey, United States) for fluorescence analysis.

### 2.7 Measurement of ROS Levels

The using DCFH-DA assay was used to measure the levels of ROS in HL-7702 cells. The cells were cultured as indicated in Section 2.2. Then, 10 μM DCFH-DA was added to the cells, followed by incubation at 37°C for 30 min in the dark. The cells were rinsed in PBS and re-suspended in the same PBS prior to injection into a flow cytometer for measurement of levels of ROS.

### 2.8 Determination of the Oxidative Stress Index

The cells were seeded in 6-well plates at a density of 4.0 × 10^5^ cells/well, and cultured for 24 h. Then, the cells were incubated with 5, 10, and 20 μM of ginsenoside Rb1 for 6 h, followed by incubation with 400 nM TP for 48 h. Thereafter, the cells were centrifuged 10 min at 12,000 rpm and lysed with radio-immunoprecipitation assay (RIPA) buffer. The protein content of the lysate was determined with CA protein quantification kit. The levels of intracellular malondialdehyde (MDA), GSH, and SOD were measured using their respective assay kits.

### 2.9 Measurement of Mitochondrial Membrane Potential (Δψm)

The assay of mitochondrial membrane potential (MMP) assay was done using JC-1. In normal cells with high mitochondrial membrane potential, a large amount JC-1 in the mitochondrial matrix produces red fluorescence. In contrast, in apoptotic cells, the reduced MMP promotes the transfer of JC-1 to the cytoplasm, where it appears green fluorescence (Luo et al., 2018). Therefore, changes in MMP can be judged by changes in fluorescence of JC-1. In this assay, the cells incubation and drug treatment methods were the same as described in Section 2.2. Thereafter, the cells were rinsed in PBS and incubated with in culture medium mixed with JC-1 staining solution at 37°C for 30 min. After washing twice with PBS, the changes in MMP were analyzed in a flow cytometer.

### 2.10 Cell Cycle Assay

The cells were incubated and treated with drugs as described in Section 2.2. After discarding the medium, the cells were digested using trypsin and washed with PBS, followed by re-suspension in 75% ethanol overnight at 4°C. Then, the cells were incubated with propidium iodide (PI) and ribonuclease (RNase) for 30 min in the dark, followed by analysis of cell cycle using a flow cytometry.

### 2.11 Western Blot Analysis

The methods used for cell culture and drug treatment were as indicated in Section 2.2. Total protein was extracted by lysing the cells with ice-cold RIPA buffer (Sigma, St. Louis, MO, United States). The lysate was centrifuged for 10 min at 12,000 rpm, and the protein content of the supernatant was determined using BCA protein assay kit. In a parallel experiment, the mitochondrial and cytosolic fractions were separated using the ProteoExtract^®^ Cytosol/Mitochondria Fractionation Kit (Millipore, Billerica, MA, United States) according to the manufacturer’s instructions. Then, equal amounts of target protein were separated using sodium dodecyl sulfate-polyacrylamide gel electrophoresis (SDS-PAGE) and transferred to a polyvinylidene fluoride (PVDF) membrane. The protein-loaded membranes were blocked with 5% skim milk in TBST buffer (25 mM Tris, 150 mM NaCl, and 0.1% Tween 20) pH 7.4 for 1 h at room temperature. Thereafter, the membrane was incubated overnight at 4°C with specific primary antibodies against cytochrome *c* (1:1,000), Bcl-2 (1:1,000), Bax (1:1,000), Fas (1:1,000), p53 (1:1,000), p21 (1:1,000), p-p53 (1:1,000), CDK2 (1:1,000), cyclin A (1:1,000), cyclin E (1:1,000), cleaved-caspase-3 (1:1,000), cleaved-PARP (1:1,000), cleaved-caspase-9 (1:1,000), Nrf2 (1:1,000), Keap1 (1:1,000), HO-1 (1:1,000), NQO1 (1:1,000), COX IV (1:1,000), Histone H3 (1:1,000), and β-actin (1:1,000). Then, the immunoblots were incubated with and corresponding secondary antibodies were incubated together at room temperature for 1 h. Finally, the relative expression levels were determined using intensive ECL detection system (UVP, United States), with β-actin and COX IV as loading controls (cytosolic and mitochondrial, respectively).

### 2.12 Co-Immunoprecipitation Assay

Following treatment with ginsenoside Rb1, the HL-7702 cells were exposed to TP for 48 h. Then, the cells were lysed using protease inhibitor-containing RIPA buffer so that Nrf2 existed in the cells in the form of Keap1/Nrf2 complex. The lysed product was incubated with corresponding primary antibody overnight at 4°C. Before being mixed with the cell lysate, protein A/G magnetic beads were washed thrice with RIPA buffer, followed by centrifugation for 3 min at 3,000 rpm. The mixture was incubated at 4°C for 4 h to ensure that the antibody was bound to the protein A/G magnetic beads. Then, 2 μg anti-Keap1 antibody or anti-Nrf2 antibody was added to the cell lysate and incubated for 24 h, after which the complex was washed 3–5 times, and boiled for 5 min in SDS sample buffer. Then, the supernatant was subjected to SDS-PAGE electrophoresis to obtain Nrf2 or Keap1 antibody immunoblot.

### 2.13 siRNA Transient Transfection

Nrf2 was silenced in HL-7702 cells by using Nrf2 siRNA. The following siRNA-encoding DNA oligonucleotides containing inner palindromic sequences (sense: 5′-CGA​GAA​GUG​UUU​GAC​UUU​ATT-3′; antisense: 5′-UAA​AGU​CAA​ACA​CUU​CUC​GTT-3′, and negative control siRNA (NC siRNA) (sense: 5′-UUC​UCC​GAA​CGU​GUC​ACG​UTT-3′; antisense: 5′-ACG​UGA​CAC​GUU​CGG​AGA​ATT-3′) were synthesized (GenePharma, United States). HL-7702 cells were transiently transfected with Nrf2 siRNA or NC siRNA by using Lipofectamine 2000 (Invitrogen, Carlsbad, CA, United States) according to the associated protocol. Moreover, at 24 h post-transfection, the cells were treated by indicated drugs and collected for Western blot analysis.

### 2.14 Statistical Analysis

All experimental data were expressed as mean ± S.D (*n* = 3). GraphPad Prism 8.0 was used to analyze the experiment data. Statistical differences were determined with one-way ANOVA and LSD test. Values of *p* < 0.05 were considered indicative of statistically significant differences.

## 3 Results

### 3.1 Ginsenoside Rb1 Inhibited Triptolide-Induced Cytotoxicity in HL-7702 Cells

**FIGURE 2:**
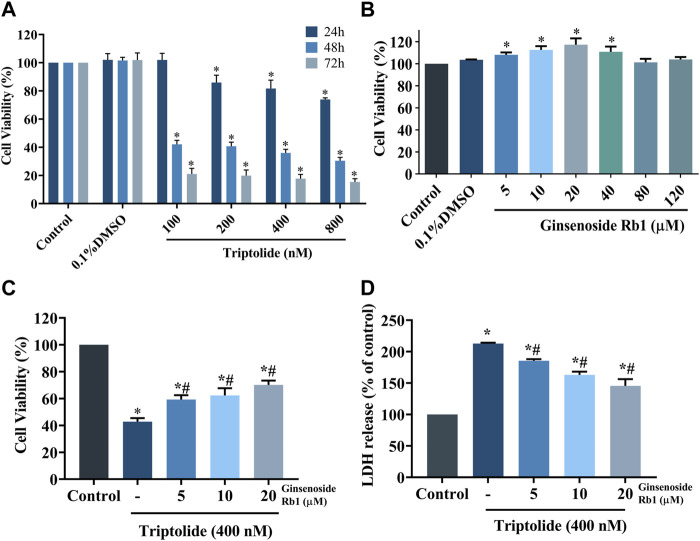
Protective effect of ginsenoside Rb1 on TP-induced cytotoxicity in HL-7702 cells. **(A)** Viability of HL-7702 cells after treatment with different concentrations of TP for different time durations, as determined with MTT assay. **(B)** Viability of HL-7702 cells treated with various concentrations of ginsenoside Rb1 for 24 h. **(C)** HL-7702 cells were pretreated with ginsenoside Rb1 for 6 h, and then exposed to 400 nM TP for 48 h, after which cell viability was determined with MTT assay. **(D)** Protective effect of ginsenoside Rb1 on TP-induced LDH release from HL-7702 cells. LDH is mainly located in the cytoplasm, and its release indicates the damage of cell membrane integrity. Data are expressed as mean ± S.D, *n* = 3. (*p < 0.05 *vs.* untreated cells in control group; ^#^ *p* < 0.05 *vs.* TP-only group).

The HL-7702 cells were exposed with 100, 200, 400, and 800 nM of TP for 24, 48, and 72 h, respectively, aim at determining the dose and treatment time of TP according to the result of cell viability using MTT assay. Compared with control group, TP dose-dependently reduced the viability of HL-7702 cells, suggesting that it exerted toxic effects on HL-7702 cells (Figure 2A). After repeated experiments, it was found that 400 nM TP treat for 48 h had stable and obvious cytotoxicity on HL-7702 cells. As a result, 400 nM TP was used as the condition in the subsequent experiments. In contrast, ginsenoside Rb1 (at concentrations of 5, 10, and 20 μM) dose-dependently enhanced the viability of HL-7702 cells (Figure 2B). Therefore, 5, 10, and 20 μM ginsenoside Rb1 were used in follow-up experiments. The HL-7702 cells were treated with ginsenoside Rb1 at doses of 5, 10, and 20 μM for 6 h, followed incubation with 400 nM TP for 48 h. Results from MTT assay showed that the viability of ginsenoside Rb1-treated cells at each of the three concentrations was markedly higher than that of the control group, indicating that ginsenoside Rb1 protected HL-7702 cells from TP-induced toxicity (Figure 2C). Results from LDH assay were consistent with those from cell viability assay: ginsenoside Rb1 reversed the TP-induced LDH release in a dose-dependent manner (Figure 2D). These results suggest that ginsenoside Rb1 dose-dependently inhibited TP-induced cytotoxicity in HL-7702 cell.

### 3.2 Ginsenoside Rb1 Inhibited Triptolide-Induced HL-7702 Cell Apoptosis

**FIGURE 3:**
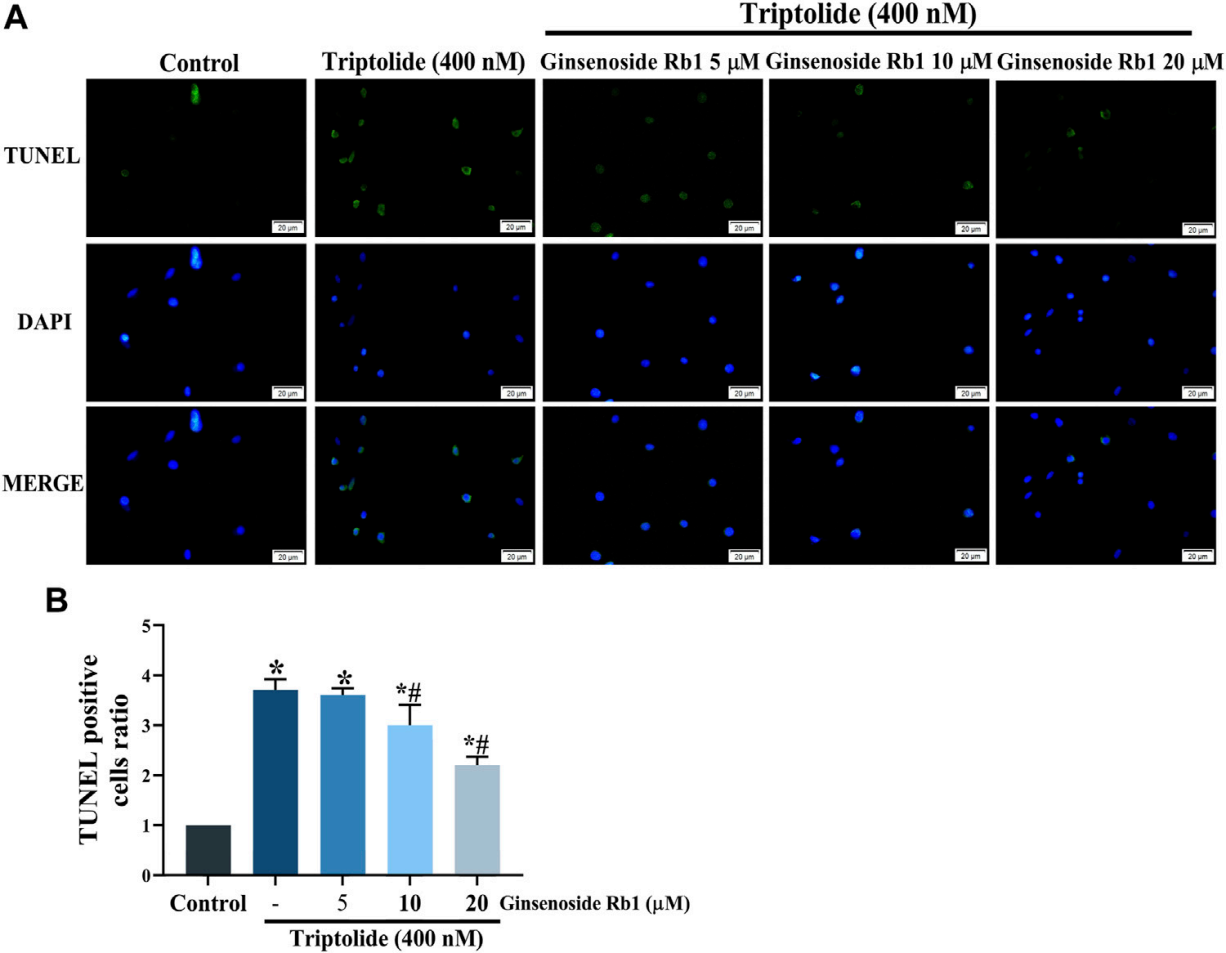
Protective effect of ginsenoside Rb1 against apoptosis in HL-7702 cells. **(A)** TUNEL staining of apoptotic cells under fluorescence microscopy (scale bar = 20 μm). TUNEL reagents were used to label the apoptotic cells and all cell nuclei were stained with DAPI. **(B)** Histogram of proportion of TUNEL positive cells. Data are expressed as mean ± S. D, *n* = 3. (*p < 0.05 *vs.* untreated cells in control group; ^#^ *p* < 0.05 *vs.* TP-only group).

**FIGURE 4:**
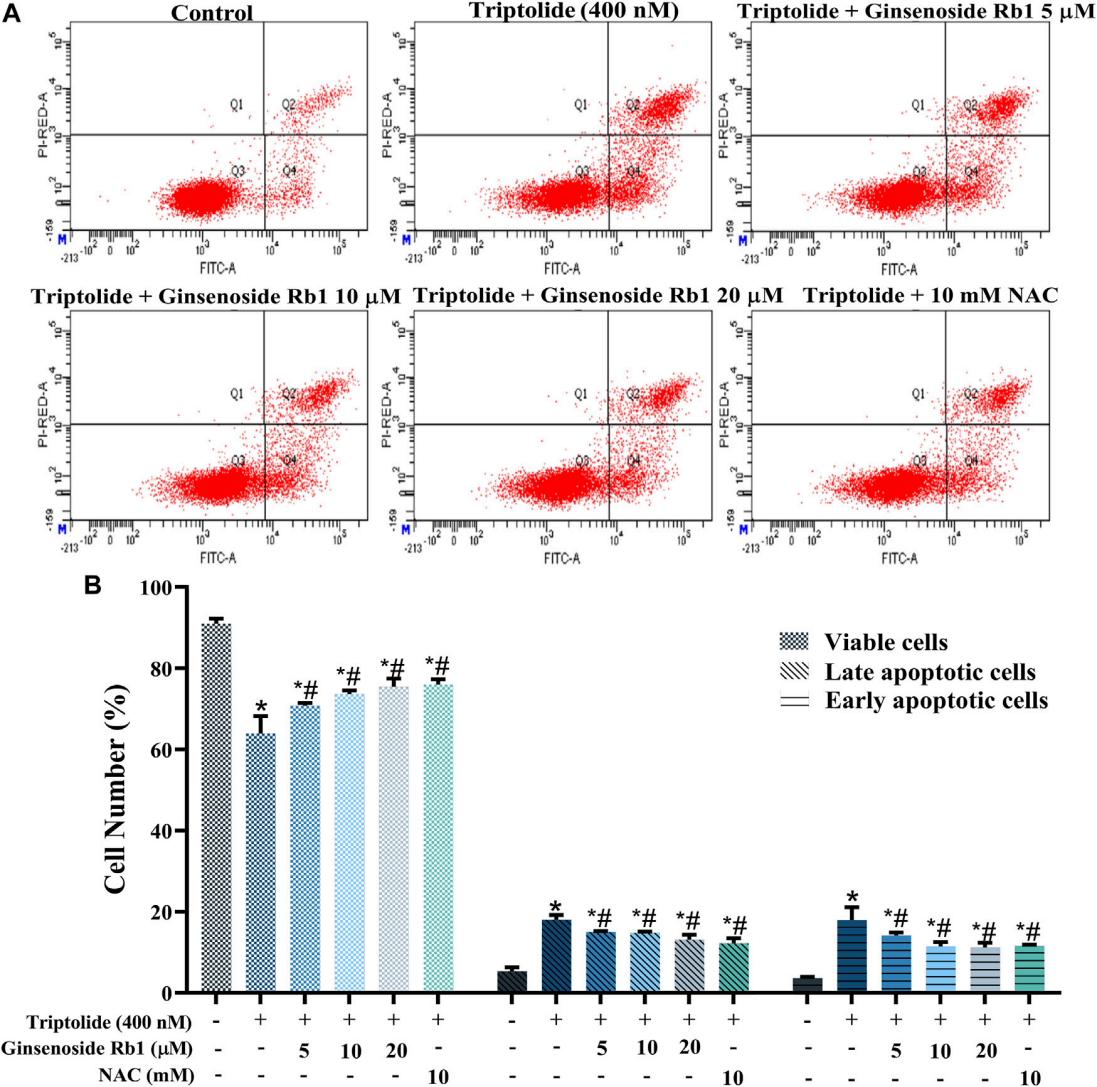
Protective effect of ginsenoside Rb1 against apoptosis in HL-7702 cells. **(A)** Apoptosis of HL-7702 cells treated with 5, 10, 20 μM ginsenoside Rb1, as determined using Annexin V-FITC/PI double staining and flow cytometry. The Q3 region represents viable cells, while Q4 region represents early apoptotic cells. The Q2 region represents late apoptotic cells, while Q1 region represents necrotic cells or mechanical damaged cells. **(B)** Histogram showing the average percentages of viable, early apoptotic, and late apoptotic cells. Data are expressed as mean ± S.D, *n* = 3. (*p < 0.05 *vs.* untreated cells in control group; ^#^ *p* < 0.05 *vs.* TP-only group).

TUNEL nuclear staining and Annexin V-FITC/PI flow cytometry were used to determine the protective effect of ginsenoside Rb1 against TP-induced apoptosis in HL-7702 cells. The percentage of apoptosis i.e., TUNEL-positive cells due to TP treatment was significantly increased (Figures 3A,B). In contrast, ginsenoside Rb1 pretreatment at doses of 5, 10, and 20 μM inhibited TP-induced apoptosis. The purpose of Annexin V-FITC/PI flow cytometry assay was to quantify the degree of apoptosis in each group of treated cells. In 5, 10, and 20 μM ginsenoside Rb1-pretreatment groups, the percentage of viable HL-7702 cells was markedly higher than that in cells treated with TP only, indicating that ginsenoside Rb1 suppressed apoptosis induced by TP (Figure 4A). Moreover, the percentages of early and late apoptotic cells were markedly increased in the TP-treated group, but the pretreatment ginsenoside Rb1 effectively reversed this change (Figure 4B). It is known that NAC, a type of ROS inhibitor, partially blocks TP-induced apoptosis. Following treatment with NAC, the percentage of viable cells was significantly increased, while the percentage of early and late apoptotic cells were decreased, which were similar to the effect of ginsenoside Rb1. These results suggested that ginsenoside Rb1 may inhibit the production of ROS, thereby inhibiting the occurrence of apoptosis (Figures 4A,B). Overall, the results indicated that ginsenoside Rb1 suppressed TP-induced HL-7702 cell apoptosis.

### 3.3 Effect of Ginsenoside Rb1 on Triptolide-Induced Cell Cycle Arrest in HL-7702 Cells

**FIGURE 5:**
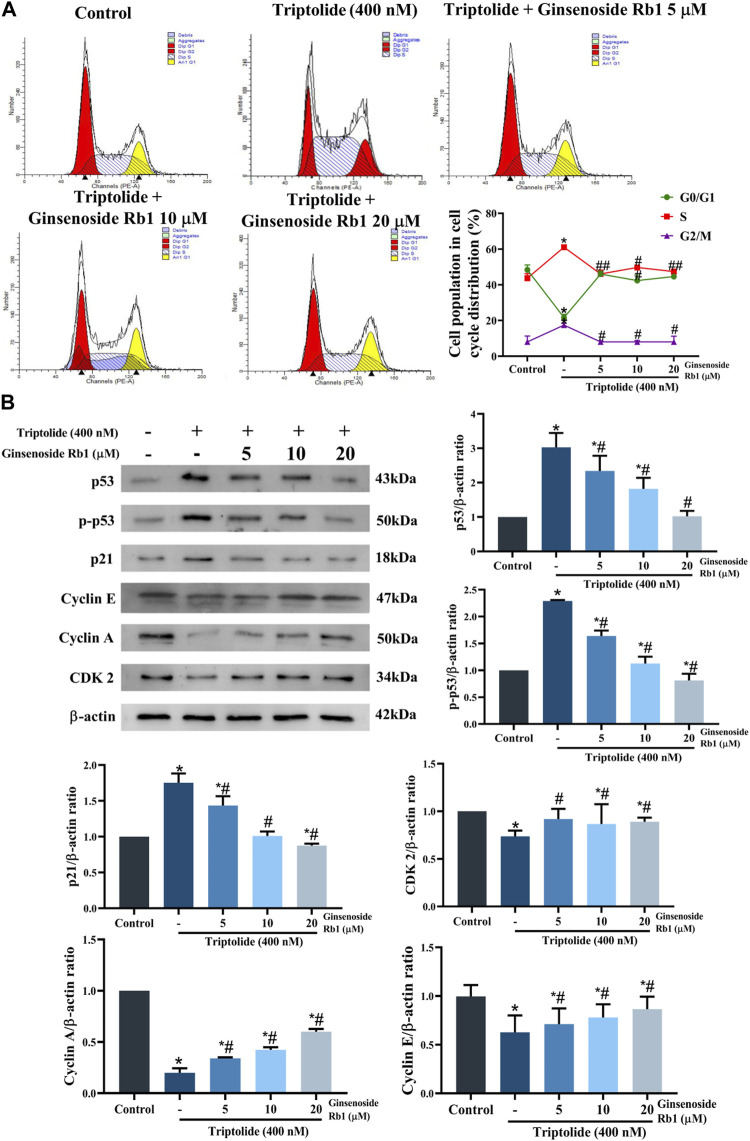
Effect of ginsenoside Rb1 on TP-induced cell cycle arrests in HL-7702 cells. **(A)** Effect of 5, 10, and 20 μM ginsenoside Rb1 on the cell cycle. **(B)** Effect of 5, 10, and 20 μM ginsenoside Rb1 on the expressions of proteins related to the cell cycle, as determined with Western blotting. Data are expressed as mean ± S.D, *n* = 3. (*p < 0.05 *vs.* untreated cells in control group; ^#^ *p* < 0.05 *vs.* TP-only group).

The effect of ginsenoside Rb1 on the cell cycle distribution in TP-treated HL-7702 cells was determined with flow cytometry. Treatment of the cells with TP led to marked increases in the percentages of cell in G2/M and S phases of the cell cycle, but the percentage of cell in the G1 phase was significantly reduced (Figure 5A). In contrast, ginsenoside Rb1 pretreatment significantly mitigated G2/M and S phase cell cycle arrest induced by TP. These results are in agreement with the expression levels of proteins related in the G2/M and S phases. In TP-treated group, the protein expression levels of p53, p-p53, and p21 were markedly increased, but the protein expressions of cyclin A, cyclin E, and CDK2 significantly downregulated (Figure 5B). However, ginsenoside Rb1 pretreatment effectively reversed the pattern of expressions of these proteins. Thus, ginsenoside Rb1 pretreatment effectively inhibited TP-induced cell cycle arrest in HL-7702 cells.

### 3.4 Effect of Ginsenoside Rb1 on the Modulation of Apoptosis-Related Proteins in Triptolide-Treated HL-7702 Cells

**FIGURE 6:**
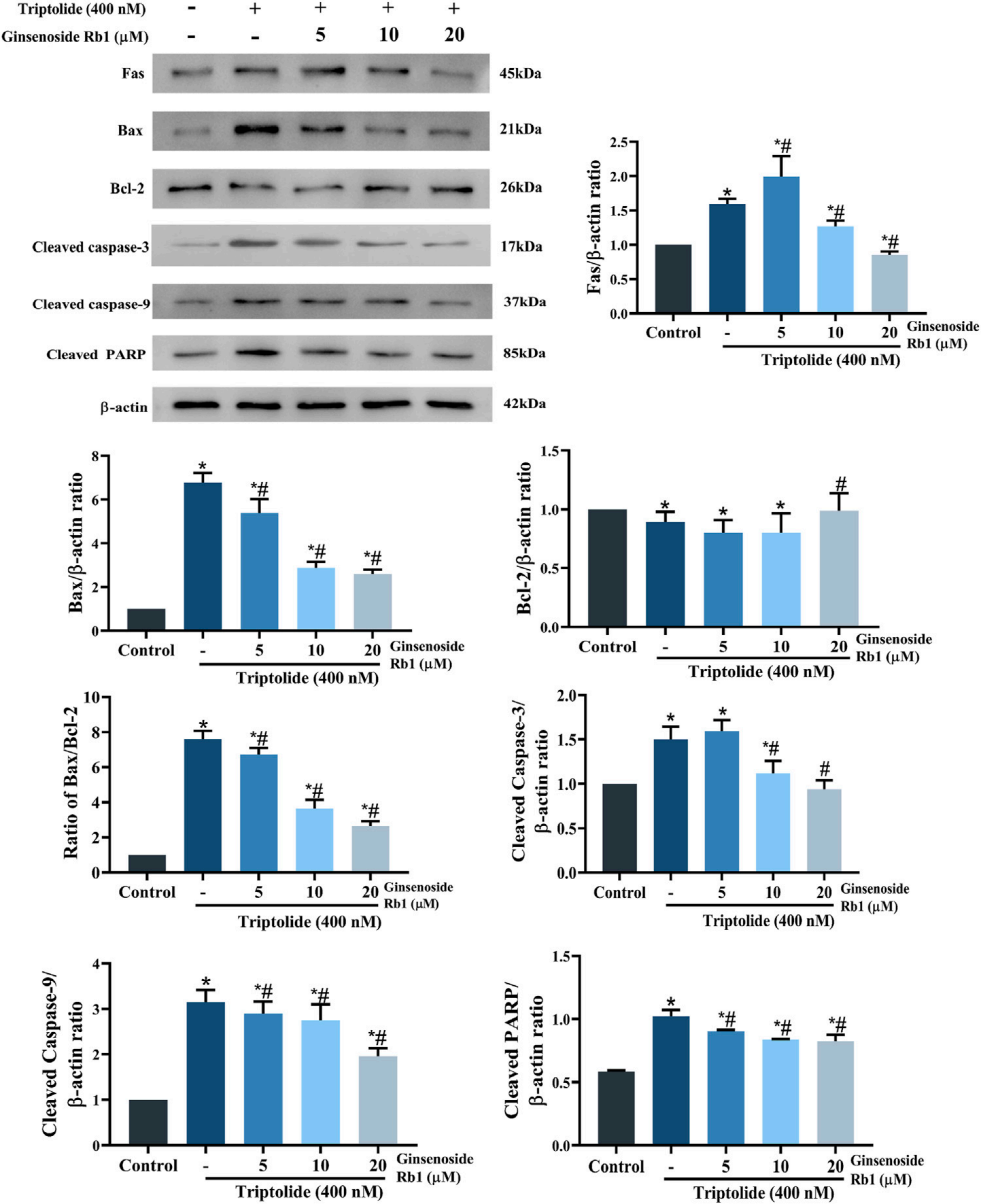
Effect of ginsenoside Rb1 on the expressions of apoptosis-related proteins in TP-treated HL-7702 cells. Data are expressed as mean ± S.D, *n* = 3. (*p < 0.05 *vs.* untreated cells in control group; ^#^ *p* < 0.05 *vs.* TP-only group).

The expressions of apoptosis-related proteins were assayed with Western blotting so as to further identify the potential regulatory mechanism involved in the effect of ginsenoside Rb1 on cell apoptosis. In the group treated with TP only, the protein expressions of Fas (one of the most typical death receptors) and Bax were significantly increased. However, these increases were reversed by ginsenoside Rb1 in a dose-dependent manner (Figure 6). Exposure of the HL-7702 cells to 400 nM TP for 48 h resulted in significant increases in the protein expressions of cleaved caspase-3, cleaved PARP, and cleaved caspase-9. However, these increases were dose-dependently reversed by pretreatment with ginsenoside Rb1. These results indicated that ginsenoside Rb1 inhibited TP-induced cell apoptosis through suppression of the activation of extrinsic and intrinsic apoptotic pathways.

### 3.5 Ginsenoside Rb1 Suppressed Triptolide-Induced Oxidative Stress Activation and Mitochondrial Dysfunction in HL-7702 Cells

#### 3.5.1 Ginsenoside Rb1 Inhibited Triptolide-Induced Oxidative Stress in HL-7702 Cells

**FIGURE 7:**
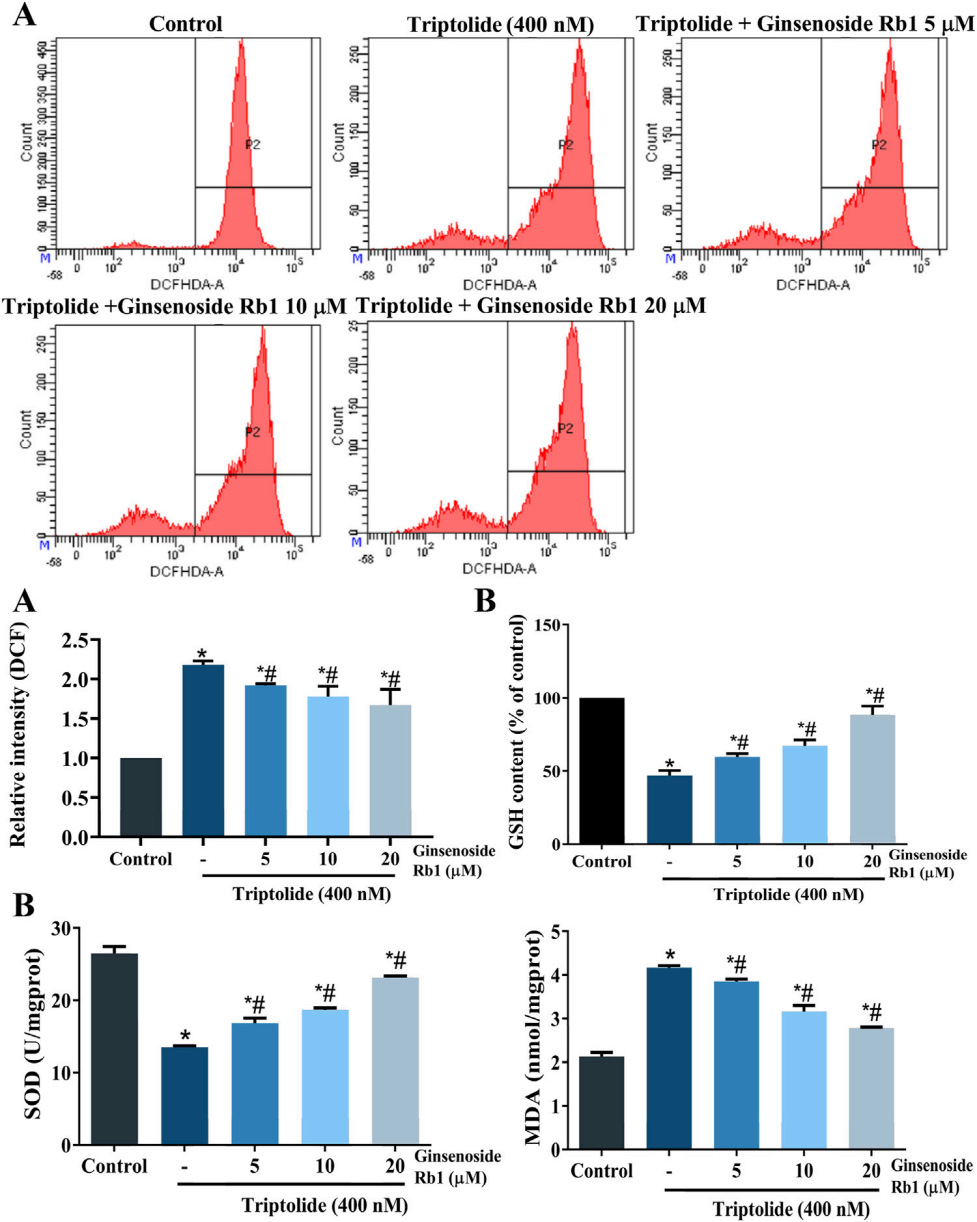
Effect of ginsenoside Rb1 on ROS levels and antioxidant enzyme activity in TP-treated HL-7702 cells. **(A)** Effects of TP and ginsenoside Rb1 on ROS levels in HL-7702 cells, as determined with DCFH-DA staining and flow cytometry. Intracellular ROS could oxidize nonfluorescent and colorless DCFH-DA into DCF, which could emit fluorsecence. Thus, fluorescence intensity coule reflect the level of intracellular ROS. **(B)** Effect of TP and ginsenoside Rb1 on levels of MDA, SOD, and GSH. Data are expressed as mean ± S.D, *n* = 3. (*p < 0.05 *vs.* untreated cells in control group; ^#^ *p* < 0.05 *vs.* TP-only group).

High levels of ROS and MDA, and reduced levels of SOD and GSH, are indicators of oxidative stress, and apoptosis (Aleksandrova et al., 2021). As expected, TP markedly increased the levels of ROS and MDA, when compared with untreated cells, while it decreased the levels of SOD, and GSH (Figures 7A,B). In contrast, the levels of intracellular ROS and MDA were decreased in ginsenoside Rb1-pretreated groups, while GSH and SOD levels were increased. These results indicated that ginsenoside Rb1 blocked TP-induced oxidative stress. Moreover, the results indicated that NAC reduced HL-7702 cell apoptosis, and demonstrated that ROS overproduction was correlated with oxidative stress-induced apoptosis (Figure 4A). Overall, ginsenoside Rb1 protected HL-7702 cells from oxidative stress-mediated apoptosis.

#### 3.5.2 Effect of Ginsenoside Rb1 on Triptolide-Induced Mitochondrial Dysfunction in HL-7702 Cells

**FIGURE 8:**
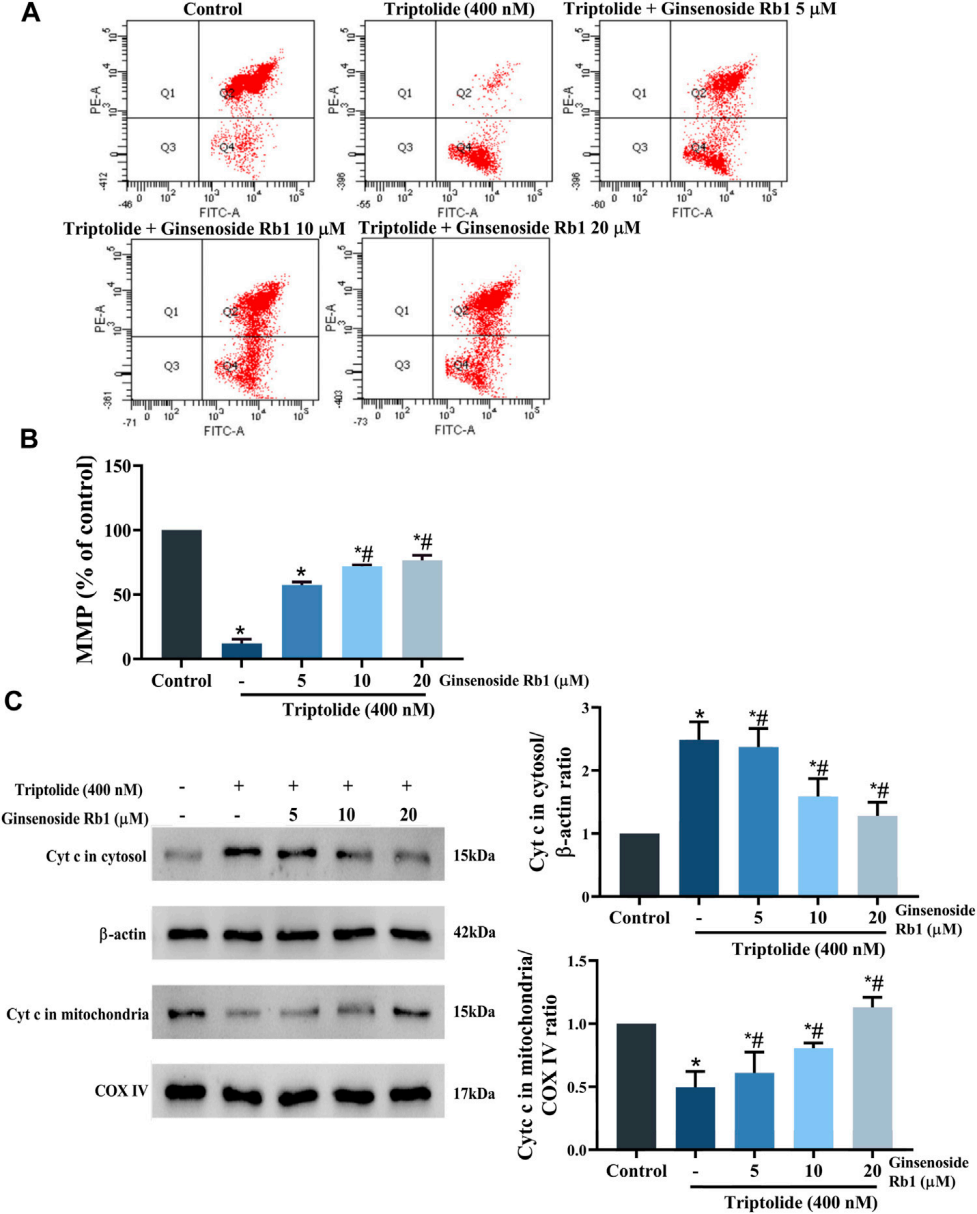
Effect of ginsenoside Rb1 on MMP in HL-7702 cells treated with TP. **(A)** Changes in MMP in the cells after treatment with TP and ginsenoside Rb1, as measured using JC-1 staining. JC-1 is a mitochondria-specific lipophilic cationic fluorescence dye able to selectively enter the mitochondria. When the mitochondrial membrane potential decreased, JC-1 transferred from the mitochondria to the cytoplasm, and the fluorescence changed from red to green. **(B)** Histogram of average JC-1 fluorescence in cells. **(C)** Effect of TP and ginsenoside Rb1 on the quantity of cytochrome *c* in the cytosol and mitochondria, as determined using Western blotting, with β-actin and COX IV as loading controls (cytosolic and mitochondrial, respectively). Data are expressed as mean ± S.D, *n* = 3. (*p < 0.05 *vs.* untreated cells in control group; ^#^ *p* < 0.05 *vs.* TP-only group).

Studies have revealed that loss of MMP increases the permeability of the mitochondrial outer membrane, resulting in severe mitochondrial dysfunction due to rapid leakage of cytochrome *c* from the mitochondria into the cytoplasm (Chen et al., 2016). Compared with untreated cells, the MMP of HL-7702 cells was markedly decreased after treatment with TP only (Figures 8A,B). However, in ginsenoside Rb1+TP groups, MMP was markedly elevated in a concentration-dependent manner. Bax (a proapoptotic protein) and Bcl-2 (an antiapoptotic protein) are the apoptosis regulatory proteins involved in the mitochondria-dependent apoptotic pathway (Jia et al., 2015). Compared with untreated cells, treatment with TP markedly enhanced Bax/Bcl-2 ratio in HL-7702 cells (Figure 6). However, pretreatment with 5–20 μM ginsenoside Rb1 effectively reversed the TP-mediated expression patterns of Bax and Bcl-2. Besides, there was marked reduction in amount of cytochrome *c* transferred to the cytoplasm in ginsenoside Rb1+TP groups, when compared to the corresponding level in cells treated with TP lone (Figure 8C). These results suggested that the mechanism underlying the effect of ginsenoside Rb1 on TP-induced apoptosis of HL-7702 cells may involve the repair of mitochondrial dysfunction.

### 3.6 Ginsenoside Rb1 Activated Keap1/Nrf2/ARE Pathway in HL-7702 Cells Under Oxidative Stress

**FIGURE 9:**
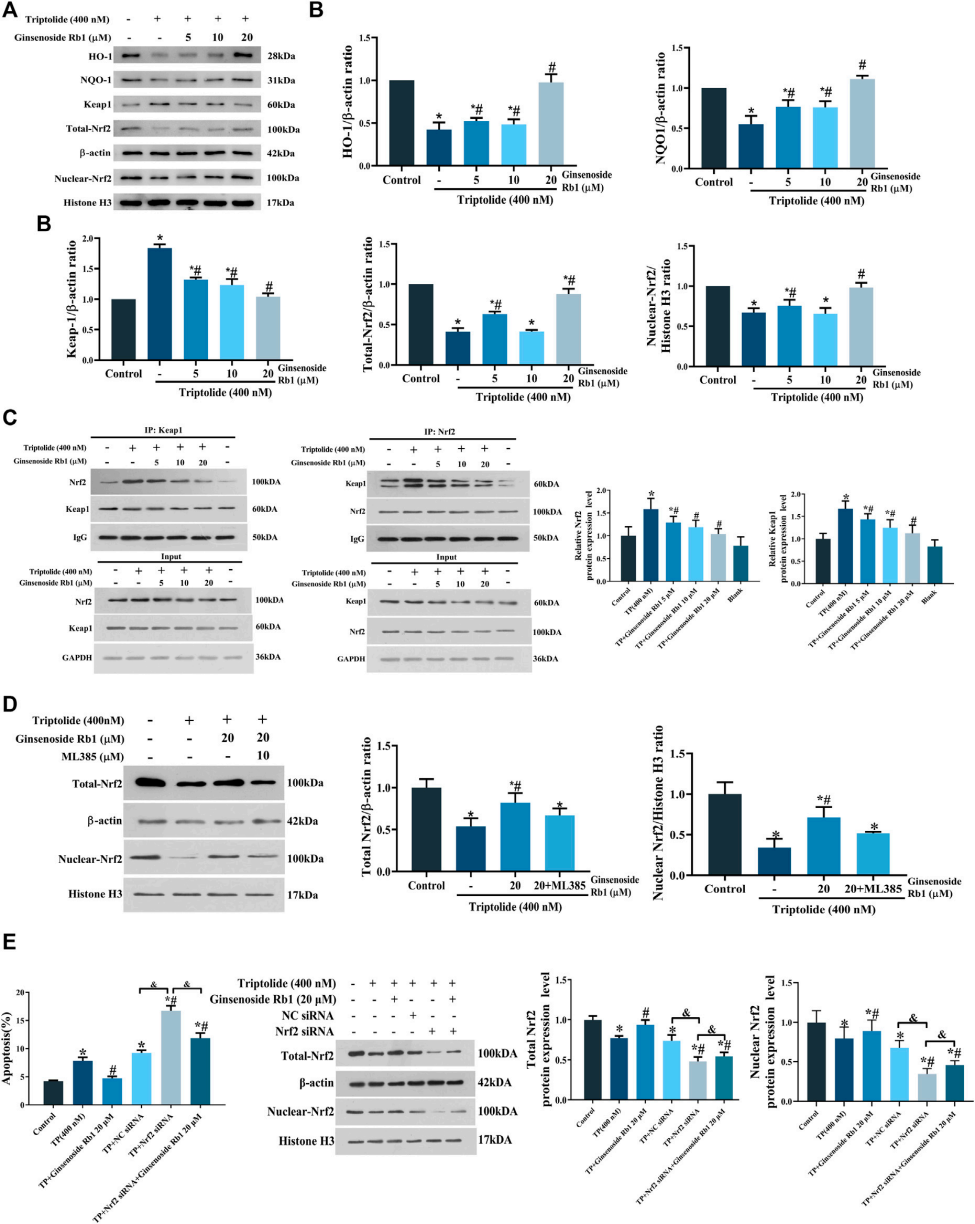
Effect of ginsenoside Rb1 on the Keap1/Nrf2/ARE pathway in HL-7702 cells treated with TP. **(A)** Effect of different concentrations of ginsenoside Rb1 on protein expression levels of Keap1, nuclear Nrf2, total Nrf2, NQO1, and HO-1, as was determined using Western blotting. Histone H3 was used as an internal control for nucleoprotein, while β-actin was used as internal control for total protein. **(B)** Quantitative protein bands of Keap1, nuclear Nrf2, total Nrf2, NQO1, and HO-1, as determined using optical density analysis. **(C)** Effect of ginsenoside Rb1 on the production of Keap1/Nrf2 complex, as determined using co-immunoprecipitation assay. **(D)** Expression levels and quantitative protein bands of Nrf2 after adding ML385 to the high-dose ginsenoside Rb1+TP group, as determind with Western blotting and optical density analysis. **(E)** The effect of Nrf2 knockdown on apoptosis and Nrf2 expression measured by flow cytometry and Western blotting. Data are expressed as mean ± S.D, *n* = 3. (*p < 0.05 *vs.* untreated cells in control group; ^#^ *p* < 0.05 *vs.* TP-only group; ^&^ *p* < 0.05 indicates significant differences between different groups).

It has been demonstrated that Nrf2 plays a central role in the cellular defense system against oxidative stress: Nrf2 activation upregulates the expressions of downstream antioxidant enzymes, especially NQO1 and HO-1 (Zhang et al., 2008). In this study, TP treatment significantly downregulated the expressions of total Nrf2, nuclear Nrf2, NQO1, and HO-1 and simultaneously increased the protein expression level of Keap1 in the cytoplasm, relative to untreated cells (Figures 9A,B). However, these TP-induced changes were significantly reversed by treatment with ginsenoside Rb1. Under regular physiological conditions, Nrf2 is rapidly ubiquitinated and degraded upon binding to Keap1, thereby inhibiting its nuclear translocation (Nguyen et al., 2009). Results from immunoprecipitation assays showed that ginsenoside Rb1 pretreatment promoted the dissociation of Nrf2/Keap1 complex in the cytoplasm (Figure 9C). In addition, the result of Nrf2 inhibitor experiment showed that ML385 could block the ginsenoside Rb1-mediated increase in the expression of total-Nrf2 protein in TP-treated HL-7702 cells (Figure 9D). What’s more, Nrf2 siRNA was used to knockdown the expression of Nrf2, and the results showed that the apoptosis of Nrf2 siRNA group was significantly higher than that of NC siRNA, while ginsenoside Rb1 effectively inhibited Nrf2 siRNA-induced apoptosis. Meanwhile, the results of WB assay showed that ginsenoside Rb1 effectively reversed the decrease of Nrf2 expression induced by Nrf2 siRNA (Figure 9E). Above all, these results indicated that ginsenoside Rb1 effectively repaired TP-induced oxidative stress damage in HL-7702 cells through activation of the Keap1/Nrf2/ARE antioxidant pathway, thereby regulating cellular oxidation-antioxidation homeostasis.

## 4 Discussion

Triptolide (TP), the main bioactive compound in TWHF, exerts significant antineoplastic, and anti-inflammatory properties. (Yang et al., 2017). However, TP is also the main component involved in the hepatotoxicity of TWHF, which greatly limits its clinical application (Zou et al., 2020; Zheng et al., 2021). Ginsenoside Rb1 is a panaxadiol saponin extracted from *ginseng*, *Panax notoginseng*, American ginseng, and other Araliaceae plants. It has been reported that ginsenoside Rb1 exerts multiple pharmacological actions such as cerebral protection, cardiovascular system protection, immunoregulation and anti-leukemia effects (Zymone et al., 2018; Fan et al., 2019; Cao et al., 2020). Although the pharmacological effects of ginsenoside Rb1 have been gradually understood over the years, its potential hepatotoxic effect (especially TP-induced hepatotoxicity), and the mechanism involved, remain unclear. Results from MTT and LDH assays indicated that TP exerted obvious cytotoxic effect on HL-7702 cells. However, HL-7702 cell viability was increased after treatment with ginsenoside Rb1, indicating, significant attenuation of TP-induced cytotoxic effect on the cells. Apoptosis is triggered by exogenous and endogenous pathways, and the caspase family plays a crucial role in both pathways (Shao et al., 2019; Wang et al., 2019b). Fas is one of the most typical death receptors in the exogenous death receptor-mediated apoptosis pathway. It recruits Fas-associated death domain (FADD) after binding to FasL (the specific ligand of Fas), thereby activating caspase-8. Then the release of caspase-8 into the cytoplasm activates caspase-3, ultimately triggering apoptosis (Xu et al., 2020). The endogenous apoptotic pathway is also called mitochondria-dependent apoptotic pathway. The Bcl-2 protein family and cytochrome *c* are involved in the mitochondria-dependent apoptotic pathway (Berg et al., 2013). Bax is one of the important pro-apoptotic proteins in Bal-2 family. The transfer of activated Bax into the mitochondria impairs mitochondrial membrane permeability and causes increased of cytochrome *c* into the cytoplasm. In the presence of ATP, cytochrome *c* forms a polymer complex with apoptosis protease activating factor-1 (Apaf-1), and then activates pro caspase-9 to form apoptotic bodies composed of cytochrome *c*, caspase-9, and Apaf-1. Subsequently, the apoptotic bodies enhance the transcription and translation of downstream proteins, especially caspase-3, eventually leading to apoptosis (Jia et al., 2015; Li et al., 2017; Chen et al., 2018). However, Bcl-2 (one of the most typical anti-apoptotic proteins) suppresses Bax activity and plays an anti-apoptotic part by inhibiting cytochrome *c* release and attenuating mitochondrial membrane damage (Xu et al., 2019c). This study has demonstrated that TP treatment markedly upregulated the expressions of cleaved caspase-3, cleaved PARP, cleaved caspase-9, and Fas, and increase the ratio of Bax/Bcl-2 in HL-7702 cells. In addition, TP induced increased release of cytochrome *c*. However, these TP-induced changes in HL-7702 cells were suppressed by pretreatment with ginsenoside Rb1. Overall, these results indicated that ginsenoside Rb1 effectively protected the HL-7702 cells from apoptosis induced by TP through inhibition of death receptor-mediated and mitochondrial-dependent apoptotic pathways.

Mitochondrial dysfunction caused by oxidative stress is an important event in apoptosis. Excessive ROS in cells is a sign of oxidative stress (Li et al., 2018; Chen et al., 2020). Slight increases in intracellular ROS levels are beneficial to cell proliferation, while excessive ROS cause structural changes in related proteins and lipid peroxidation, resulting in mitochondrial dysfunction which is manifested in increased mitochondrial membrane permeability, decreased mitochondrial membrane potential, and increased cytochrome *c* release (Chen et al., 2016; Kong et al., 2018). SOD and GSH are typical antioxidants, which inhibit oxygen free radical production and scavenge lipid peroxides produced by ROS (Song et al., 2018). In this study, TP markedly increased intracellular ROS levels and Bax/Bcl-2 ratio in HL-7702 cells, and reduced the MMP and levels of SOD and GSH, while the MDA level was increased. However, ginsenoside Rb1 pretreatment reversed the TP-induced increases in levels of ROS and MDA, and increased the levels of SOD and GSH. Moreover, it was observed that NAC pretreatment markedly reversed TP-induced apoptosis in HL-7702 cells. In general, these results have demonstrated that ginsenoside Rb1 effectively inhibited TP-induced oxidative stress and promoted the antioxidant capacity of HL-7702 cells.

The Nrf2 pathway is considered to be an important route for combating oxidative damage caused by excessive ROS (Liu et al., 2018). Normally, Nrf2 exists in the cytoplasm in the form of Keap1/Nrf2 complex. When the levels of intracellular ROS increase, Nrf2 binds to ARE in the nucleus, thereby upregulating the expressions of HO-1, and NQO-1 (Yang et al., 2020). In this study, TP markedly reduced the levels of total Nrf2 and nuclear Nrf2, while ginsenoside Rb1 effectively reversed these changes. Moreover, ginsenoside Rb1 suppressed the production of Nrf2/Keap1 complex. Addition of ML385 to the high-dose ginsenoside Rb1+TP group blocked the increase of the total Nrf2 expression. In addition, Nrf2 siRNA was used to knockdown the expression of Nrf2 in HL-7702 cells. The results indicated that ginsenoside Rb1 could significantly inhibit the apoptosis of HL-7702 cells and reverse the decrease of Nrf2 expression induced by Nrf2 siRNA. Thus, ginsenoside Rb1 effectively activated antioxidant defenses through stimulation of the Keap1/Nrf2/ARE pathway. Pretreatment with ginsenoside Rb1 triggered the transcription and translation of downstream antioxidant proteins such as NQO-1, and HO-1, indicating that it played an antioxidant role. The activation of Keap1/Nrf2/ARE signaling pathway appears to be one of the crucial mechanisms through which ginsenoside Rb1 protected HL-7702 cells from TP-induced oxidative damage.

Studies have shown that oxidative stress may cause DNA damage and cell cycle arrest (Samikkannu et al., 2015). It has been reported that p53 mediates oxidative stress in cells with irreparable DNA damage, and induces apoptosis in concert with other apoptotic proteins e.g., Bax (Hori et al., 2013). The combination of cyclin A and CDK2 or CDK1 regulates S phase and mitosis (Wang et al., 2019b). Cyclin E-CDK2 complex regulates the duplication of intracellular chromosomes which promote the entry of cells into the S phase, and on entering the G2 phase, there is a gradual decrease in cyclin E content (Shang et al., 2020). Moreover, p21 is one of the most typical cyclin-dependent kinase inhibitors. By binding to cyclin and CDK, p21 inactivates the cyclin-CDK complex, and blocks each stage of the cell cycle (Lee et al., 2017). In this study, the HL-7702 cells were arrested at G2/M and S phases after treatment with TP, and TP upregulated the expressions of p21, p-p53, and p53, while downregulated the expressions of cyclin A, cyclin E and CDK2. However, these expression patterns were effectively reversed by ginsenoside Rb1 pretreatment. These results indicated that excessive ROS production caused DNA damage, and further led to arrest of HL-7702 cells at G2/M and S phases of the cell cycle, and mitochondria-dependent apoptosis. Therefore, it can be hypothesized that ginsenoside Rb1 protected HL-7702 cellular DNA from oxidative damage by inhibiting ROS production, thereby indirectly reversing G2/M and S cell cycle arrest while inhibiting endogenous apoptosis pathway.

## 5 Conclusion

The results of this study have shown that ginsenoside Rb1 protects HL-7702 cells from TP-induced apoptosis and cell cycle arrest via activation the Keap1/Nrf2/ARE pathway. This study has reported, for the first time, that ginsenoside Rb1 can alleviate TP-induced cytotoxicity in HL-7702 cells, and its potential mechanism is elucidated from the perspective of combination medication, providing new strategies for clinical treatment of TP-induced hepatotoxicity.

## Data Availability

The original contributions presented in the study are included in the article/Supplementary Material, further inquiries can be directed to the corresponding authors.
